# Treatment of Pathologic Proximal Femur Fractures Using the Improvised Megaprosthesis: Combination of the Hip Prosthesis and Intramedullary Nail

**DOI:** 10.5704/MOJ.2503.012

**Published:** 2025-03

**Authors:** DK Carolino, AR Tud

**Affiliations:** Musculoskeletal Tumor Service, Philippine Orthopedic Center, Quezon City, Philippines

**Keywords:** endoprosthetic replacement, improvised megaprosthesis, kuntscher nail, pathologic fracture, proximal femur

## Abstract

**Introduction:**

The proximal femur is the most common long bone affected by metastatic disease. Pathologic fractures in this area are frequent, secondary to weight-bearing and deforming forces. Long-stem endoprosthetic replacement is often used to replace and bypass segments affected by metastases. However, implant cost remains prohibitive for patients in low-resource settings. An improvised megaprosthesis using a hip implant combined with Kuntscher nail provides an economic option.

**Material and Methods:**

This is a case series of three patients diagnosed with pathologic fracture of the hip secondary to metastatic bone disease who underwent proximal femoral resection with reconstruction using an improvised endoprosthesis in a single tertiary hospital. Outcomes determined include total blood loss, total surgical time, length of hospital stay, latest functional score using the Musculoskeletal Tumour Society (MSTS) score, and pain scale using the numerical rating scale (NRS).

**Results:**

For case 1, a 42-year-old female with metastatic breast carcinoma, currently alive with disease and able to perform activities of daily living (ADLs) with minimal assistance; for case 2, a 77-year-old male diagnosed with prostatic carcinoma, able to ambulate with assistive device before expiring 2 years post-surgery; and for case 3, a 57-year-old female with metastatic breast carcinoma, able to resume unassisted ADLs at 3 months post-surgery before refusing systemic treatment in her second year of surveillance monitoring.

**Conclusion:**

An improvised megaprosthesis is a cost-effective implant option in low-resource settings, which may help decrease complications related to immobilisation for patients undergoing palliative surgery for metastatic bone disease.

## Introduction

The burden of cancer continues to increase worldwide, with metastatic bone disease (MBD) among its most common complications. The femur is the most frequently involved long bone by skeletal metastases, with the proximal third being a particular site of concern for pathologic fractures. Fifty percent of these occur at the femoral neck, 30% in the subtrochanteric area, and 20% along the intertrochanteric line^1-3^.

Palliative limb salvage is the current standard of care for treating metastatic bone lesions. A long-stem endoprosthesis is preferred for proximal femur lesions to provide intramedullary fixation and in anticipation of progressive diaphyseal involvement^2-3^.

However, modular, intercalary, and large segment endoprosthesis are difficult to procure for patients with limited financial capacity in developing countries. Delays secondary to unavailable implants often result in progressive bone destruction and additional morbidity, further decreasing quality of life for patients. To address these concerns, our institution has adopted the use of an improvised tumour endoprosthesis created by combining a standard femoral stem with a Kuntscher nail to span the femoral diaphysis.

Similar techniques have been described by only two case series previously by Lim *et al* (2019) and Richards *et al* (2010)^4,5^. This is the first case series describing the technique and outcomes for Filipino patients with pathologic hip fractures secondary to MBD.

## Materials and Methods

This is a case series of 3 patients (Table I) diagnosed with a primary malignancy with pathologic fracture of the hip secondary to MBD. They all underwent proximal femoral resection in a single tertiary hospital.

**Table I:** Summary of surgical and functional outcomes of cases.

	Primary disease	Blood loss	Surgical time	Latest follow-up post-surgery	MSTS score	Pain scale (NRS)	Current status
Case 1: 42/F	Invasive ductal carcinoma, right breast	900 cc	6 hours	15 months	28/30	0-1/10	Stable disease
Case 2: 77/M	Prostatic carcinoma	1,700 cc	4.75 hours	12 months	12/30	2-3/10	DOD
Case 3: 57/F	Invasive ductal carcinoma, right breast	700 cc	2.75 hours	14 months	12/30*	7-8/ 10*	DOD

*Due to progression of metastasis to contralateral femur

**Fig. 1:**
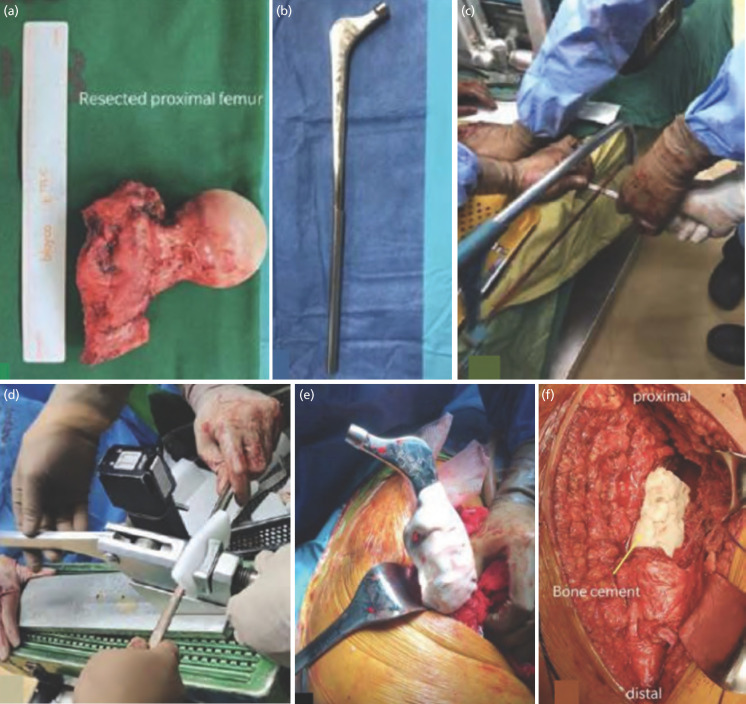
(a) Gross picture of the resected proximal femur. (b) Improvised megaprosthesis fashioned from a smooth bipolar femoral stem prosthesis inserted into the opened end of a Kuntscher nail, which was shortened using a hacksaw (c) after the desired length was calculated. (d) The opened end of the Kuntscher nail was then crimped closed around the end of femoral stem. (e) Cementation followed by implantation of the prosthesis. (f) Reduction of the hip after cement has set.

Posterior approach to the hip was used for all patients, which involved detachment of the gluteus medius and short external rotators. Proximal femoral resection was performed (Fig. 1a) according to the measured length of affected femur on the available imaging. The improvised megaprosthesis was assembled by opening the Kuntscher nail (sized accordingly to patient’s isthmus) and slotting in the end of a smooth stemmed femoral stem (using either a bipolar or an Austin-Moore prosthesis) with a minimum length of 3cm (Fig. 1b). The excess length of the Kuntscher nail was cut with a hacksaw (Fig. 1c). The previously opened segment of the nail was then crimped to tighten around the end of the stem (Fig. 1d). Reaming of the intramedullary canal was performed up to 1.5mm more than the estimated diameter of the Kuntscher nail. Cement was placed into the distal femoral canal using a cement gun, followed by prosthetic implantation, with at least 13cm of femoral implant embedded within bone to ensure stability (Fig. 1e). After setting of the bone cement, the hip is reduced (Fig. 1f) and the capsule is tightened around the prosthesis. The vastus lateralis was then sewn to the mesh over the prosthesis, including the remaining psoas, gluteus, and external rotators. Subsequent wound closure was done in layers.

Patients were referred for rehabilitation a day immediately after surgery for general conditioning and strengthening. Range of motion exercises were initiated with instructions to avoid hip flexion beyond 90°, to avoid adduction beyond midline, and to keep rotation of the limb in neutral. Patients are allowed transfers to wheelchair and gait retraining with full weight bearing as tolerated.

Outcomes determined include total blood loss, total surgical time, length of hospital stay, latest functional score using the Musculoskeletal Tumour Society (MSTS) score, and pain scale using the numerical rating scale (NRS). Approval was obtained from the local ethics committee and institutional review board.

## Results

**Fig. 2:**
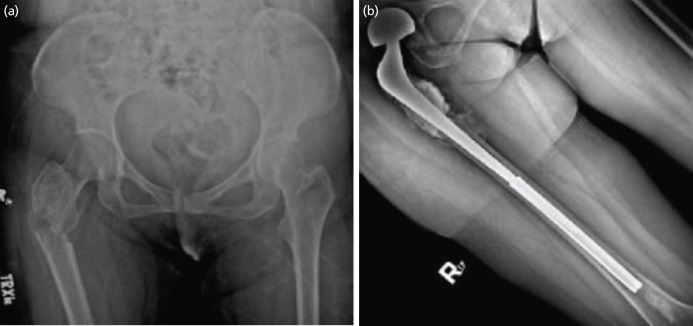
(a) Injury radiograph of the pelvis in AP view, with an intertrochanteric fracture in the right proximal femur and note of a lytic lesion in the area of the per trochanteric area. Bone scan later on revealed presence of metastatic lesions in the diaphysis of the right femur. (b) Post-operative radiograph of the femur in AP view at latest follow-up at one year and three months, with no signs of dislocation, recurrence or implant failure observed.

For case 1, a 42-year-old female diagnosed with invasive ductal carcinoma of the right breast slipped and fell from standing height. Severe pain at the right hip with inability to ambulate prompted consult. Radiographs were consistent with a pertrochanteric pathologic fracture of the right proximal femur (Fig. 2a). Patient was referred to Medical Oncology and subsequently completed six cycles of chemotherapy. Whole body bone scan revealed multiple metastases on the skull, left humeral head, ribs, multiple vertebrae, iliac regions, and ipsilateral femoral shaft. She underwent proximal femoral resection followed by reconstruction with a bipolar prosthesis attached to a Kuntscher nail. Total surgical time lasted 6 hours, with an estimated blood loss of 900cc. No perioperative complications were noted. On the 9th day after surgery, the patient was able to ambulate with partial weight bearing on the right using a walker. The patient was sent home 10 days post-surgery. MSTS score at 15 months post-surgery (Fig. 2b) was 28, with an NRS of 0-1/10. She has since received bisphosphonate (zoledronic acid 4mg/5ml) once a month post-operatively. At present she is able to perform all activities of daily living with minimal assistance only when donning footwear.

**Fig. 3:**
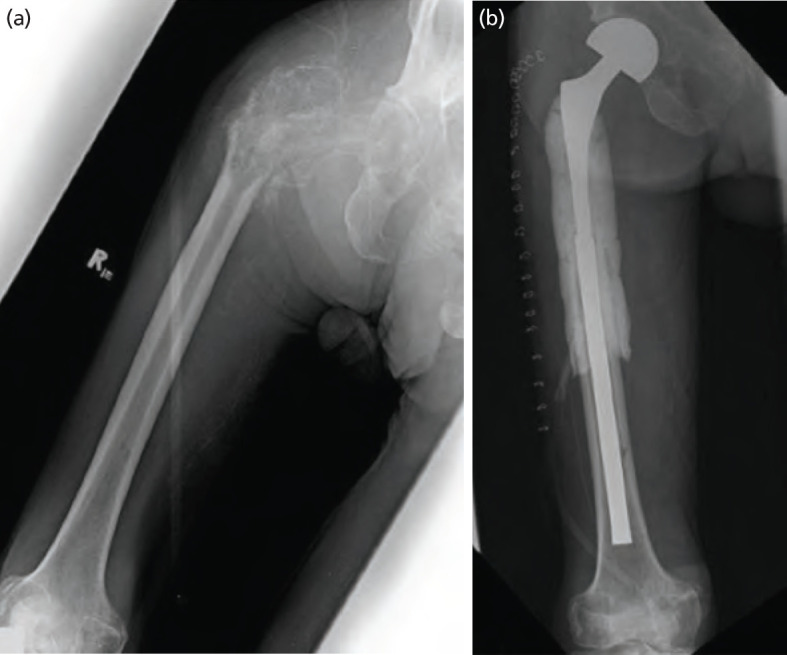
(a) Injury films of the right femur in AP view, revealing a fracture and a lytic lesion in the per trochanteric area of the right proximal femur. (b) Post-operative radiograph at one year and two months, with no signs of dislocation, recurrence, or implant failure.

For case 2, a 77-year-old male slipped and fell from standing height, sustaining a pertrochanteric pathologic fracture of the right proximal femur. Diagnostic tests confirmed multiple metastatic lesions throughout the length of the right femur and right iliac bone (Fig. 3a). Further investigation revealed history of difficulty urinating and prostate specific antigen showed elevated levels (>100ng/mL). A biopsy done via transurethral resection of the prostate confirmed a prostatic carcinoma. The patient underwent proximal femoral resection followed by reconstruction using a cemented bipolar hip prosthesis attached to a Kuntscher nail. Total surgical time was 4.75 hours and estimated blood loss was 1,700cc. No complications were documented during the post-operative period. Patient was able to tolerate wheelchair transfers six days after surgery and was discharged the day after. He refused further chemotherapy, radiotherapy, or bisphosphonates. At 12 months post-surgery (Fig. 3b), the patient was able to ambulate with assistance at home, with an MSTS score of 12 and NRS of 2-3/10. Following a second fall reported by family members 14 months after surgery however, his health rapidly declined. The patient was reportedly dead of disease (DOD) 22 months post-surgery.

**Fig. 4:**
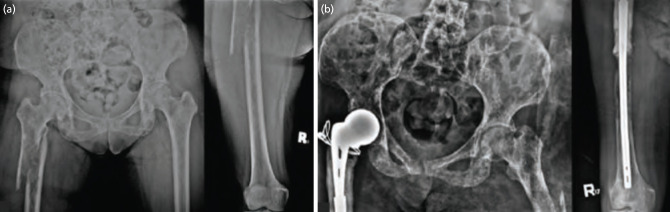
(a) Injury films of the right femur in AP view, revealing a fracture in the subtrochanteric area of the right proximal femur and a lytic lesion extending in its metadiaphyseal areas. (b) Post-operative radiographs at latest follow-up at two years, with no signs of dislocation or implant failure, but with note of progression of the lytic lesions in the pelvis and further distal to the previously applied cement, in addition to the diaphyseal fracture on the contralateral femur.

For case 3, a 57-year-old female diagnosed with invasive ductal carcinoma of the right breast reported severe pain while ascending stairs, followed by inability to ambulate. Radiographs confirmed a pathologic subtrochanteric fracture at the right proximal femur (Fig. 4a). Diagnostic imaging revealed multiple lytic foci in the skull, humerii, right femoral shaft, and multiple vertebrae. She underwent proximal femoral resection followed by cemented partial hip replacement using an Austin-Moore prosthesis attached to a Kuntscher nail (Fig. 4b). Total surgical time was 2.75 hours with an estimated blood loss of 700cc. She was able to sit at bedside on the 3rd day and was discharged on the 11th day post-operatively. By one month, she was able to do assisted wheelchair transfers. At three months post-surgery, the patient was able to resume work as a hairdresser without assistance but did not follow-up for radiotherapy, systemic, or bisphosphonate treatment. At 12 months post-surgery, MSTS score was 21 and NRS of 3/10. However, at 14 months after surgery, declining function with an MSTS of 12 was reported due to contralateral thigh and low back pain, with a NRS of 7-8/10. At 23 months post-surgery, she sustained a fracture on the contralateral femur after alighting from a car. Further work-up revealed progression of metastasis, including pulmonary and hepatic involvement. She refused further treatment and is currently DOD.

## Discussion

Advances in early detection and initiation of treatment contributes to longer life expectancy among cancer patients, with a resultant increase in incidence of MBD. Pathologic fractures secondary to metastatic lesions often indicates the terminal stage of a primary malignant tumour^3^. Authors have reported variable survival rates after surgery, ranging from 16% to 30% at one year, 7% to 29% at two years, and 4.5% to 11% at three years and beyond^1,3^. The wide range of survival rates have been attributed to varying baseline health status and cancer stage^3^.

Pathologic fractures of the proximal femur result in severe pain and immobility, which may lead to complications known to reduce survival. These include pneumonia, bedsores, deep vein thrombosis, and urinary tract infections^2,3^. Surgical options include endoprosthetic reconstruction, closed reduction with intramedullary nailing, and open reduction with internal fixation. The choice of fixation technique depends on the location of the lesion, presence of a fracture, amount of bone involvement, primary tumour, patient’s life expectancy and personal preference, and response to treatment^3^.

A study by Yu *et al* in 2017^3^ investigated 88 cases to determine clinical, functional, and oncologic outcomes following endoprosthetic replacement (EPR) versus intramedullary nailing (IMN) in patients with metastic disease of the proximal femur. All patients demonstrated significantly higher MSTS scores at six weeks compared to their pre-operative scores, with no significant difference between the two groups at three months post-operatively. Local recurrence rates were lower in the EPR group at 10.5%, as compared to the IMN group at 25.8%, however the difference was not significant. On the other hand, complication rate in the IMN group was 29% and was significantly higher than the EPR group at 10.5%.

While non-union and nail breakage were the primary causes of implant failure in their study, authors reported that the excessive cost of EPR still makes IMN a viable option, particularly in the context of comparable functional outcomes and palliative patient status^3^.

In 2019, Lim *et al* first described the use of an Austin Moore prosthesis secured to a Kuntscher nail with cerclage wire for patients with pathologic fractures of the proximal femur, citing the need for a more cost-effective alternative to EPR^4^. All six patients in their series were started on wheelchair ambulation in the immediate post-operative period, with one patient able to ambulate as early as six days after surgery, and no report of dislocations. Mean survival period post-surgery was 3.9 months, emphasising that in this patient population, affording relief of pain and return to mobility in a short amount of time should take precedence over implant longevity^4^.

A case series by Richards *et al*^5^ described four patients for whom a highly polished stem cemented inside or alongside a Kuntscher nail was used. All four had a history of recurrent infections after total hip replacement, with concomitant severe metadiaphyseal bone loss. Mobilisation as early as the 6th post-operative day was achieved, and all patients remained pain-free with no recurrence of infection at one to three years of follow-up. With their findings, the authors concluded that their modified hip prosthesis was a viable less-expensive alternative to EPR^5^. While the population in this study is not similar to our series, it shows that this construct is applicable in cases with severe femoral osseous defects such as that secondary to MBD and has shown good outcomes in regard to ambulation status post-surgery.

At present, the local cost of the standard proximal femur tumour endoprosthesis is estimated to be 300,000 PHP (5,200 USD). On the other hand, this improvised implant is around half that cost at 150,000 PHP (2,600 USD). This underlines the consideration of a cost-efficient option in a low-resource location, especially in the context of a palliative setting and prognosis of patients afflicted with metastasis, while providing pain relief and expected functionality.

## Conclusion

For patients who are on palliative setting living in low-resource locations diagnosed with pathologic fractures of the proximal femur, reconstruction with an improvised endoprosthesis composed of a partial hip implant combined with a Kuntscher nail is a viable cost-effective option compared to the standard tumour endoprosthesis. Authors have reported relief of pain and comparable return to function in similar case series involving patients with pathologic proximal femur fractures. Larger prospective studies may be beneficial to determine short- and long-term outcomes.
